# Effect of pulmonary arterial hypertension on the morphology and antioxidant defence of the ventral prostate of sedentary and exercised rats

**DOI:** 10.1111/iep.70007

**Published:** 2025-10-21

**Authors:** Luiz Otávio Guimarães‐Ervilha, Thainá Iasbik‐Lima, Leôncio Lopes Soares, João Victor Leles Faria, Mariana Souza Oliveira, Renner Philipe Rodrigues Carvalho, Mônica Morais‐Santos, Antônio José Natali, Mariana Machado‐Neves

**Affiliations:** ^1^ Departamento de Biologia Geral Universidade Federal de Viçosa Viçosa Brazil; ^2^ Departamento de Educação Física Universidade Federal de Viçosa Viçosa Brazil; ^3^ Departamento de Biologia Animal Universidade Federal de Viçosa Viçosa Brazil

**Keywords:** inflammation, mast cells, nitric oxide, oxidative stress, superoxide dismutase

## Abstract

Pulmonary arterial hypertension (PAH) is a chronic disease characterized by increased pulmonary vascular resistance, right ventricular overload and systemic repercussions, including disorders in non‐target organs. This study aimed to investigate the effects of PAH on the ventral prostate of adult Wistar rats, as well as the role of resistance training (RT) in modulating potential changes caused by the disease. Male rats (*n* = 32, 60 days old) were divided into four groups: sedentary control, sedentary PAH, RT control and PAH + RT. PAH was induced using two injections of monocrotaline, while rats were submitted to the RT protocol for a month. Afterward, the ventral prostate was collected and analysed for biometric, histopathological and oxidative parameters (CEUA 38/2021). The results showed that PAH significantly reduced the body and prostate weights and increased glandular epithelium and stroma proportions, besides causing epithelium atrophy and inflammatory infiltrates (*p* < .05). The activity of superoxide dismutase and catalase was lower, culminating in high levels of malondialdehyde and carbonyl proteins (*p* < .05). The exercise mainly influenced biometric and stereological parameters. The RT protocol minimized the negative effect of PAH regarding catalase activity, epithelium/lumen proportion and inflammatory infiltrate incidence. However, it was ineffective in restoring prostate weight and completely normalizing markers of oxidative stress. In conclusion, PAH induces significant morphofunctional changes in the ventral prostate, including oxidative damage and tissue remodelling. Although RT exerts protective effects, its benefits are limited, highlighting the need for complementary therapies to counteract PAH‐induced prostate alterations fully.

## 1 INTRODUCTION

Pulmonary arterial hypertension (PAH) is a chronic and progressive disease with complex pathogenesis, characterized by an abnormal increase in the pressure within pulmonary arteries (< 15 mmHg), obliging the heart to work harder to pump blood through the lungs.^1^ After a long‐term period, blood overload may cause hypertrophy and eventual failure of the right ventricle, contributing to the high mortality associated with PAH.^2^ The PAH incidence varies between 2.4 and 7.6 cases per million inhabitants per year, with an estimated prevalence of 15–30 cases per million people, with a tendency to increase.^3^ The condition tends to impact the individual's health and quality of life significantly and may have implications for organs that are not the primary PAH targets.

Experimental studies have been focussed on organs involved in the PAH pathogenesis, once the lungs and heart suffer directly from the consequences of increased pulmonary artery pressure. However, there is a growing interest in investigating the systemic effects of PAH, particularly its damage on peripheral organs indirectly affected by cardiovascular dysfunction and chronic inflammatory processes mediated by PAH, such as the kidneys, liver, testis and ovaries.^4^ The relationship between PAH and non‐target organs is still poorly understood. For instance, there is no information about the interaction between PAH and prostate morphofunctionality, a sexual accessory gland that plays a vital role in sperm fertility and sexual health. Recently, we reported an impairment of the testes and epididymides in PAH rats.^5^ Thus, it is crucial to understand prostate conditions in a hypertensive environment. The prostate produces approximately 20–30% of seminal fluid that forms the semen, aiding sperm nutrition and transport until the female reproductive tract. Its proper functioning depends on precise regulation by androgenic hormones, particularly testosterone.^6^

Furthermore, the prostate is a highly vascularized organ, which makes it sensitive to changes in blood flow and blood pressure.^7^ Therefore, systemic diseases that affect circulation, such as PAH, can alter prostate morphology and function. Studies have shown that systemic arterial hypertension conditions and heart failure are associated with atrophy, decreased glandular secretion and inflammatory processes in the prostatic gland.^8^, ^9^, ^10^, ^11^ These changes can not only compromise reproductive function but also increase the risk of developing benign prostatic hyperplasia and prostate cancer.^9^

Physical exercise is widely recognized for its benefits in chronic disease treatments, including PAH conditions.^12^, ^13^, ^14^ Under controlled hypertension, physical exercise can improve aerobic capacity, increase exercise tolerance and reduce pulmonary artery pressure.^12^ Moreover, regular physical exercise positively modulates the cardiovascular and respiratory systems, improves endothelial function and stimulates nitric oxide release, a potent vasodilator that displays antioxidant and anti‐inflammatory activities.^15^ These benefits are essential for reducing systemic inflammation and oxidative stress, two processes strictly associated with PAH progression.^16^ Nevertheless, the effects of physical exercise on the prostate of PAH animals have not yet been elucidated. Preliminary studies suggested a positive influence of physical exercise on prostate health by modulating redox balance, reducing oxidative stress and attenuating deleterious processes in glandular function.^16^, ^17^ Particularly, resistance training (RT) has shown beneficial effects in maintaining the structural integrity of muscle tissues and improving the antioxidant functions of organs affected by PAH.^14^, ^18^ There is a significant gap in understanding how RT could impact the prostate under PAH conditions, which involve circulatory and inflammatory disorders.

Given the importance of investigating the effect of PAH on the prostate, as well as the potential benefits of RT in such conditions, this study aimed to evaluate the morphology and function of the ventral prostate in PAH‐monocrotaline‐induced Wistar rats and the modulatory role of RT in the glandular parameters. By exploring these relationships, this study seeks to contribute to the understanding of the systemic effects of PAH and physical exercise on the health of the ventral prostate, providing information for future therapeutic interventions aimed at preserving reproductive health in individuals with cardiovascular disease.

## 2 MATERIALS AND METHODS

### 2.1 Animals and ethics statement

This work is part of a comprehensive study investigating the effects of monocrotaline‐induced PAH on male reproductive parameters.^5^ Male Wistar rats (*Rattus norvegicus albinus*, *n* = 32, 60 days old, weighing ~200 g) were provided by the Central Animal Facility of the Center for Biological and Health Sciences of the Universidade Federal de Viçosa (UFV). The animals were housed in polypropylene cages under controlled temperature (22°C) and photoperiod (12–12‐h light/dark cycle). The animals had free access to feed and drinking water. This study was carried out by the recommendations of the National Council for the Control of Animal Experimentation (CONCEA). All experimental procedures were reviewed and approved by the UFV Animal Ethics Committee (CEUA process number 38/2021).

### 2.2 Experimental design

**FIGURE 1:**
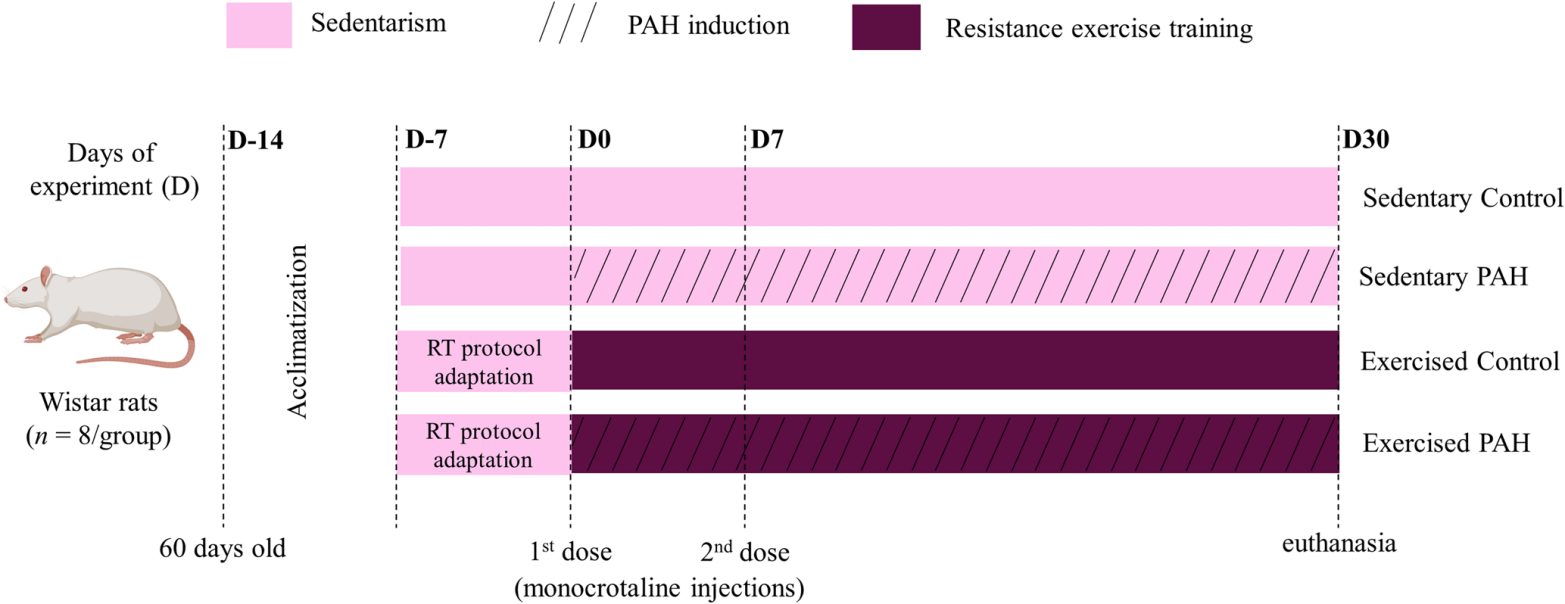
Experimental design showing the resistance training (RT) protocol and induction of pulmonary arterial hypertension (PAH). After 7 days of acclimatization, 16 animals from the control exercise and PAH exercise groups (*n* = 8/group) were subjected to the RT protocol for 7 days for adaptation, while the remaining rats from the sedentary control and sedentary PAH groups (*n* = 8/group) were not exercised. On experimental Day 0 (D0), the PAH‐induced animals received the first dose of monocrotaline (20 mg Kg^‐1^ intraperitonially), while the healthy animals received saline. On D7, the second drug application occurred. On D30, the animals were euthanized for collection of the ventral prostate.

The animals were randomly divided into four experimental groups (*n* = 8 per group; Figure 1): sedentary control, sedentary PAH, RT control, and PAH subjected to RT (PAH + RT). The 16 animals in the RT groups underwent the RT protocol for 1 week to adapt to the training protocol. Sixteen rats in the PAH groups received monocrotaline (20 mg Kg^−1^, intraperitoneal [IP]) diluted in 0.5 mL of saline (140 mM NaCl; pH 7.4) twice for PAH induction. The first injection was given on Day 0 of the experimental period, and the second dose was 7 days later (Figure 1). The monocrotaline‐induced lung injury model in rodents results in severe PAH^13^ and mimics group 1 pulmonary hypertension, according to the clinical classification of the disease.^19^, ^20^ Data on the success of PAH induction were published in our previous studies.^5^ The other 16 healthy rats from the sedentary and RT control groups received 0.5 mL of saline (IP) alone to simulate injection stress.

### 2.3 Maximum load test and resistance training

Animals from all groups adapted to a resistance training (RT) protocol as described by Hornberger and Farrar.^21^ In summary, the rats were familiarized with the RT protocol for 1 week, which consisted of climbing stairs (1.1 × 0.18 m, 2 cm spacing between the steps, 80° incline) with devices attached to their tails, without any load. Initially, the animals were encouraged to climb by applying a stimulus (pinching of the tail) to initiate movement. At the top of the stairs, there was a cage (20 × 20 × 20 cm) where they rested for 60 s. These procedures were performed five times a week.

After adapting to the RT protocol, all animals in the experimental groups underwent a maximum load test before PAH induction, and on Days 14, 21, and 28 after PAH induction. The test consisted of an initial load of 75% of body weight, which was progressively increased by 15% of body weight in subsequent climbs (120‐s intervals between each climb) until the animal could no longer climb.^13^ The maximum transport load was used as an index of physical effort tolerance.

After the first maximum load test and the first monocrotaline injection, animals subjected to exercise were incorporated into the RT program five times a week for 4 weeks. The training intensity was set at 55%–65%, calculated from the exercise tolerance test, following recommendations for patients with cardiovascular diseases. Each training session consisted of 15 climbs with 60‐s intervals between each climb. The training load was adjusted after tolerance tests conducted on Days 14 and 21 following the first monocrotaline injection.^13^

### 2.4 Euthanasia and prostate collection

On the 31st day of the experiment, the rats were weighed and sacrificed by decapitation. The ventral prostate (*n* = 8/group) was removed and dissected. After weighing, the ventral prostate was divided in half; one part was frozen in liquid nitrogen and stored at −80°C for enzymatic analysis. The other fragment was immersed in 10% buffered formalin for histopathological and morphometric analyses.

### 2.5 Assessment of antioxidant enzymes and oxidative stress markers

Ventral prostate samples (*n* = 6/group) stored at −80°C (~100 mg) were homogenized in 1 mL of phosphate buffer (pH 7.4) and centrifuged at 10,000 *g* for 10 min at 4°C. The supernatants were used to evaluate the activity of superoxide dismutase, catalase and glutathione S‐transferase, besides the quantification of by‐products yielded from nitrosative and oxidative stress, respectively, nitric oxide and malondialdehyde, and total protein quantification. The resulting pellets were used to determine carbonylated proteins. All analyses were performed using an ELISA microplate reader (Multiskan SkyHigh; Thermo Scientific).

#### 2.5.1 Superoxide dismutase

Superoxide dismutase (SOD) activity was determined by the enzyme's ability to catalyse the reaction of superoxide (O_2_^·−^) into hydrogen peroxide (H_2_O_2_), according to the auto‐oxidation of pyrogallol.^22^ The reaction mixture contained 10 μL of the sample and 170 μL of sodium phosphate buffer (pH 7.8). The reaction was initiated by adding 20 μL of pyrogallol (10 mM) for 30 min at 37°C. The reaction was measured by absorbance at 320 nm. The results of SOD activity were expressed as a unit (U)/mg of protein, with one U of SOD defined as the amount that inhibited the rate of pyrogallol auto‐oxidation by 50%.

#### 2.5.2 Catalase

Catalase (CAT) activity was assessed by measuring the rate of H_2_O_2_ decomposition, following the method described by Aebi.^23^ Briefly, 100 μL of H_2_O_2_ (20 mM) was added to 5 μL of the sample. After 3 min, 150 μL of ammonium molybdate (32.4 mM) was added to stop the reaction. Sample blanks were made by replacing H_2_O_2_ with sodium phosphate buffer (50 mM, pH 7.4). The test values were subtracted from the blank values. A standard curve was made to calculate the CAT value with the serial dilution of H_2_O_2_. The reading was performed at 374 nm in the spectrophotometer. CAT activity was expressed in U/mg of protein, with one U of CAT activity defined as the amount of enzyme that decomposes 1 mmol of H_2_O_2_ during 1 min.

#### 2.5.3 Glutathione S‐transferase

Glutathione S‐transferase (GST) activity was measured using the method of Habig et al.,^24^ considering the formation of glutathione conjugated to 2,4‐dinitrochlorobenzene (CDNB). Thus, 0.1 M CDNB and 0.1 M GSH were added to sodium phosphate buffer (0.1 M; pH 7.2) and 10 μL of the sample. After CDNB addition, the change was monitored with absorbance at 340 nm for 60 s in two intervals (30 and 90 s). The molar extinction coefficient used for CDNB was *ɛ*340 = 9.6 mmol/L × cm. GST activity was expressed in U/mg of protein, with one U of GST activity defined as the amount of enzyme that catalysed the formation of one μmol of product/min/mL.

#### 2.5.4 Nitric oxide

Nitric oxide (NO) production was determined by the Griess reaction, as described by Tsikas.^25^ Samples were incubated (50 mL) with an equal volume of Griess reagent (50 mL; 1% sulfanilamide, 0.1% N‐(1‐naphthyl)ethylenediamine, and 2.5% H_3_PO_4_) at room temperature for 10 min. The result was expressed as NO concentration (μmol/L) and was determined from a sodium nitrite standard curve (0–125 μM), with absorbance measured at 570 nm.

#### 2.5.5 Malondialdehyde

Lipid peroxidation was determined by quantifying total malondialdehyde (MDA) according to Buege and Aust.^26^ Briefly, 200 μL of the supernatant was added to 400 μL of TBARS solution (15% trichloroacetic acid, 0.375% thiobarbituric acid and 0.25 M HCl). The reaction was left for 40 min in a water bath (90°C). After cooling on ice, 600 μL of butyl alcohol was added. Then, the solution was shaken and centrifuged for 5 min at 3500 rpm. A standard curve of known concentrations of 1,1,3,3‐tetramethoxypropane (TMPO) was used to determine the MDA concentration. The formation of thiobarbituric acid‐reactive substances was monitored at 535 nm. The results were expressed as nmol/mg protein.

#### 2.5.6 Carbonylated proteins

Protein oxidation was measured by the method of Levine et al.^27^ by quantifying carbonyl groups in the protein structure. Carbonylated protein (CP) concentration was determined based on carbonyl groups reacting with 2,4‐dinitrophenylhydrazine (DNPH). The pellets resulting from sample centrifugation were added to 0.5 mL of DNPH solution (10 mM) diluted in 2 M HCl, vortexed, and kept at room temperature in the dark, shaking periodically for 30 min. Then, 0.5 mL of ice‐cold 10% TCA was added to the tubes, centrifuged (5000 *g* for 10 min at 4°C) and the supernatant was discarded. The precipitate was washed three times with 1 mL of ethyl acetate and ethanol (1:1 v/v) and centrifuged. Subsequently, 1 mL of 6% sodium dodecyl sulfate (SDS) was added and centrifuged. The supernatant was measured at 370 nm. The results were expressed as nmol/mL based on the molar extinction coefficient of ϵ370 = 22 mmol/L × cm.

#### 2.5.7 Total protein

Total protein quantification was performed using the Bradford method,^28^ using bovine serum albumin as a standard. The result was used to normalize the results of SOD, CAT, GST and MDA.

### 2.6 Histological processing and analysis

Fixed fragments of the ventral prostate were dehydrated in increasing ethanol series and embedded in 2‐hydroxyethylmethacrylate (Historesin^®^; Leica Microsystems, Nussloch, Germany). Sections of 3 μm were obtained in semi‐series, using one in every 10 sections, to avoid repeated analyses of the same histological area. The sections were stained with haematoxylin and eosin (HE) for histopathological and stereological analyses. Other sections were stained with toluidine blue for mast cell quantification and with the Picrosirius method for collagen quantification. After the staining process, the slides were mounted with Entellan (Merck, Germany). Histological fields and histological image acquisition were performed using a photomicroscope (Olympus BX53, Tokyo, Japan) connected to a digital camera (Olympus DP73, Tokyo, Japan).

The HE‐stained sections were examined and scored for benign tissue lesions, such as hyperplasia, inflammation/prostatitis, and atrophy. They were identified based on the morphological criteria described by Campolina‐Silva et al.^29^ The sections were also analysed to quantify the relative proportion of the prostatic parenchyma components: epithelium, lumen and stroma. To this end, a grid with 266 intersections was inserted over 10 random images of the prostatic parenchyma of each animal. The images were obtained using a bright‐field microscope (Olympus BX‐53, Tokyo, Japan) with a digital camera (Olympus DP73, Tokyo, Japan). The intersections totalled 2660 points per animal. The percentage of points in each component was calculated using the formula: volumetric proportion (%) = (number of points in the structure of interest/2660 total points) × 100.^30^

To quantify the mast cells present in the ventral prostate stroma, sections previously stained with toluidine blue were analysed. The dye allows the distinction of heparin cytoplasmic granules and glycosaminoglycans present in mast cells by metachromasia. Per animal, 20 histological fields were photographed using a microscope (Olympus BX53, Tokyo, Japan) connected to a digital camera (Olympus DP73, Tokyo, Japan). The images were captured randomly with a 400× magnification. The number of mast cells was quantified in each photomicrograph, and the result was normalized to 1 mm^2^.^31^, ^32^

The proportion of collagen was determined in Picrosirius‐stained sections using the ImageJ software (National Institute of Health, USA). Histological sections (*n* = 6 animals/group) were analysed, and images were captured (10 histological fields/slice, 400× magnification). The system was programmed to recognize red and green colours, estimating the percentage of total collagen in the histological field.^33^

### 2.7 Statistical analysis

The normality of the results was assessed using the Shapiro–Wilk test. Data were subjected to a two‐way analysis of variance (ANOVA) to detect the influence of two different categorical independent variables (PAH and RT) on testicular parameters (categorical dependent variables). We analysed the main effect of each independent variable and the possible interaction between RT and PAH, considering differences of *p* < .05 significant. ANOVA *p*‐values are shown in Table S1. Graphics were generated using the GraphPad Prism 8.0 software (GraphPad Software Inc., San Diego, CA, USA). Results are expressed as mean ± standard deviation (SD).

## 3 RESULTS

### 3.1 Body and organ biometry

**FIGURE 2:**
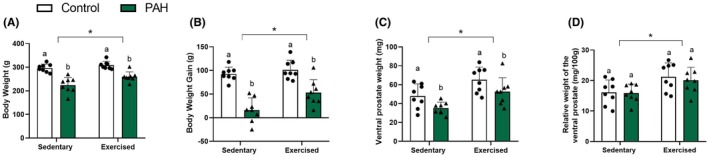
Body weight, mass gain and biometry of the ventral prostate of healthy male Wistar rats (control) and rats with PAH induced with monocrotaline submitted to resistance training (RT; exercised) or not (sedentary). Mean ± SD. ^a,b^Significant difference (*p* < .05) between control and PAH groups by two‐way ANOVA. Asterisk (*) indicates an RT effect (*p* < .05). *n* = 8 animals/group.

Animals with PAH showed lower body weight and body mass gain than healthy rats (*p* < .05; Figure 2A). Similarly, exercised rats showed higher body weight and mass gain than sedentary animals (*p* < .05; Figure 2A,B). The ventral prostate weight was lower in hypertensive animals and non‐exercised rats than in their respective controls (*p* < .05; Figure 2C). While PAH did not affect the relative prostate weight (*p* > .05; Figure 2D), the RT protocol increased the relative prostate weight in exercised rats compared to sedentary animals (*p* < .05; Figure 2D). The exercise did not influence any animal response against the negative effects of PAH regarding body and ventral prostate biometry (*p* > .05; Figure 2).

### 3.2 Antioxidant enzyme activity and nitrosative/oxidative stress markers

**FIGURE 3:**
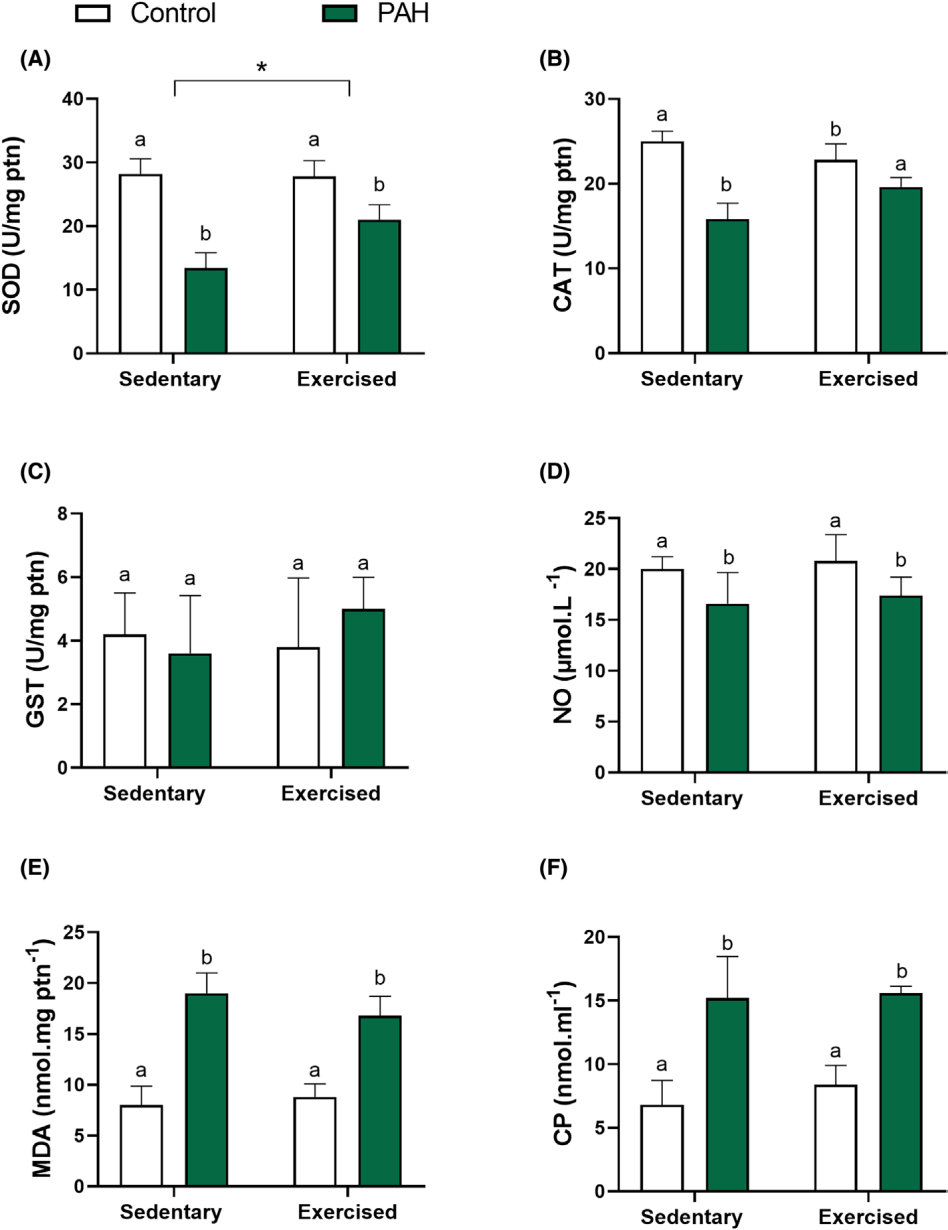
The activity of antioxidant enzymes and oxidative stress markers in the ventral prostate of healthy (control) Wistar rats and monocrotaline‐induced PAH rats subjected to resistance training (RT; exercised) or not (sedentary). CAT, catalase; CP, carbonylated protein; GST, glutathione S‐transferase; MDA, malondialdehyde; NO, nitric oxide; SOD, superoxide dismutase. Mean ± SD. ^a,b^Significant difference (*p* < .05) between control and PAH groups by two‐way ANOVA. If the letter sequence differs between sedentary and RT groups, it indicates an interaction between PAH and RT. Asterisk (*) indicates an RT effect (*p* < .05). *n* = 6 animals/group.

The activity of antioxidant enzymes in the ventral prostate was impaired under hypertensive conditions. The prostatic activity of SOD and CAT was lower in PAH rats than in healthy animals (*p* < .05; Figure 3A,B), with no impact on GST activity (*p* > .05; Figure 3C). RT practice increased SOD enzyme activity in exercised rats compared to sedentary animals (*p* < .05; Figure 3A). Moreover, the exercise attenuated the reduction of CAT activity in the rat ventral prostate under hypertensive conditions (*p* < .05; Figure 3B). Regarding by‐products of nitrosative stress, NO levels were lower in the ventral prostate of hypertensive rats than in healthy animals (*p* < .05; Figure 3D). On the other hand, the levels of the oxidative stress markers MDA and PC were higher in the prostatic tissue of PAH rats than in healthy animals (*p* < .05; Figure 3E,F). Animals submitted to the RT protocol did not present alterations in the levels of NO, MDA and PC in the ventral prostate when compared to sedentary rats (*p* > .05; Figure 3D–F). Finally, RT practice was not effective in protecting the organ against damage caused by PAH (*p* > .05).

### 3.3 Histopathology

**FIGURE 4:**
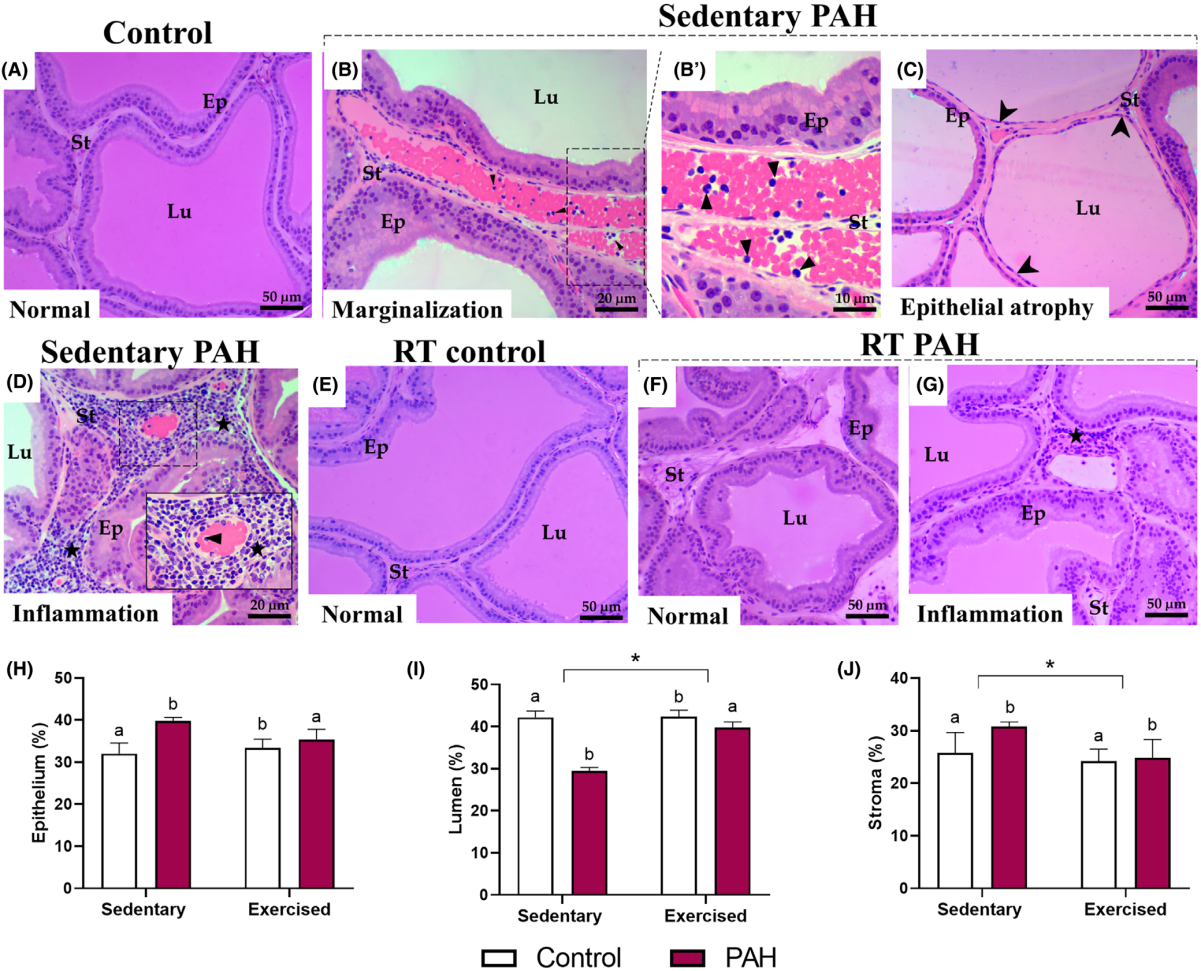
Histological sections and histomorphometric analysis of the ventral prostate from healthy Wistar rats (control) and monocrotaline‐induced PAH rats subjected to resistance training (RT; exercised) or not (sedentary). Arrowhead, epithelial atrophy; Ep, prostatic epithelium; Lu, lumen; St, stroma; Star, inflammatory infiltrate; Triangle, leukocyte marginalization. Staining: Haematoxylin and eosin. ^a,b^Significant difference (*p* < .05) between control and PAH groups by two‐way ANOVA. If the letter sequence differs between sedentary and RT groups, it indicates an interaction between PAH and RT. Asterisk (*) indicates an RT effect (*p* < .05; *n* = 6 animals/group).

In healthy rats, the ventral prostate parenchyma presented a histological architecture such as typical tubuloalveolar glands. Their acini presented slightly folded mucosa formed by simple cuboidal epithelium with secretory and basal cells. They were surrounded by a layer of smooth muscle and stroma composed of connective tissue, fibroblast cells and blood vessels (Figure 4A). Hypertensive rats, in turn, presented morphological changes in their prostatic parenchyma, such as epithelium atrophy (Figure 4C), inflammatory infiltrates in the stroma and blood vessels interspersed with defence cells, indicating leukocyte marginalization (Figure 4B,B′,D). Physical exercise did not alter the prostate histology, with acini composed of simple cuboidal epithelium and regular stroma (Figure 4E). Hypertensive rats submitted to the RT protocol showed fewer pathologies, such as less incidence of epithelium atrophy and interstitial inflammation foci (Figure 4F,G).

### 3.4 Histomorphometry and stereology

**FIGURE 5:**
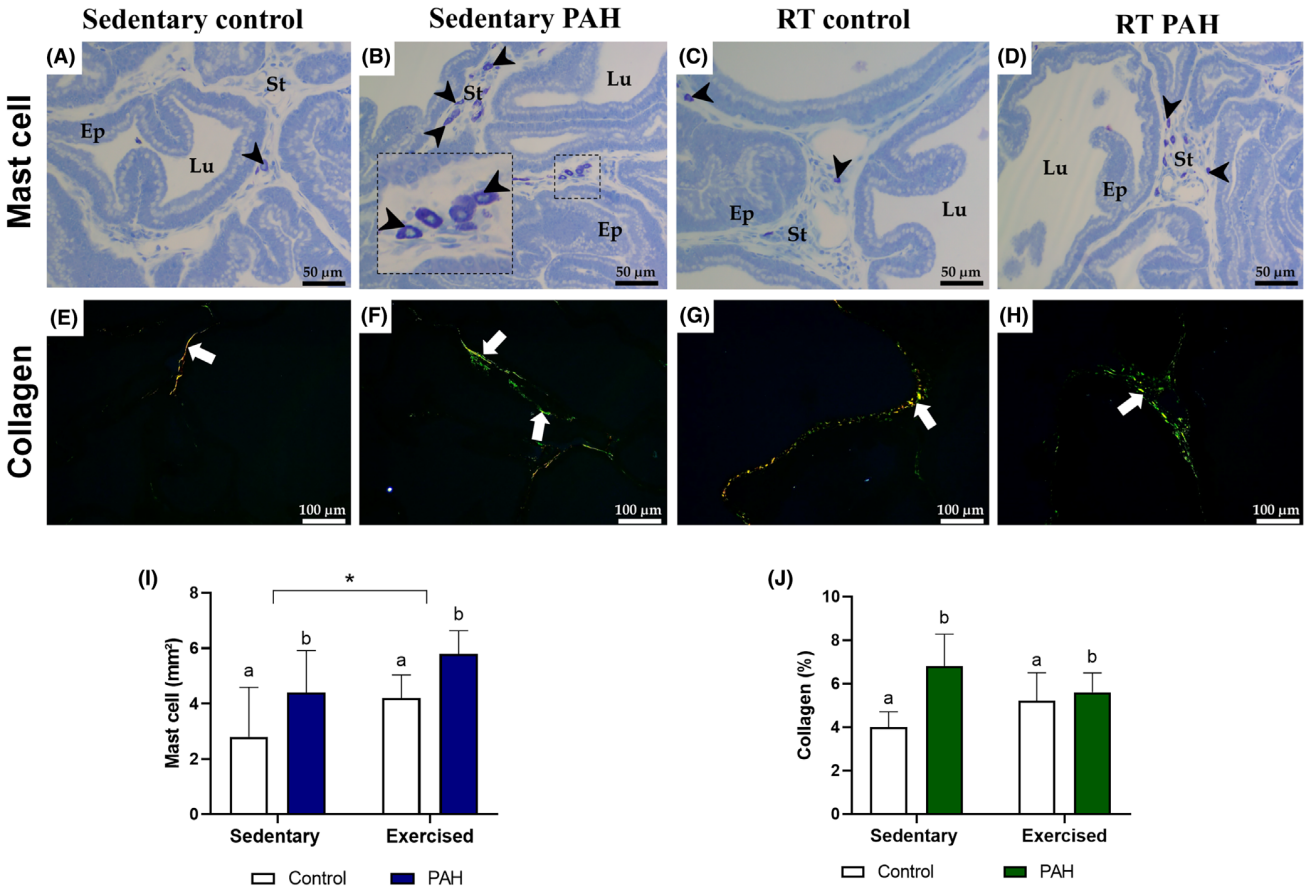
Histological sections of ventral prostate from healthy Wistar rats (control) and rats with monocrotaline‐induced PAH subjected to resistance training (RT; exercised) or not (sedentary). Arrowhead, mast cells; Ep, prostatic epithelium; Lu, lumen; St, stroma; White arrow, collagen fibres. (A–D) modified toluidine blue; (E–H) Sirius red. ^a,b^Significant difference (*p* < .05) between control and PAH groups by two‐way ANOVA. If the letter sequence differs between sedentary and RT groups, it indicates an interaction between PAH and RT. Asterisk (*) indicates an RT effect (*p* < .05; *n* = 6 animals/group).

Stereological analyses of the ventral prostate parenchyma revealed a higher proportion of epithelium and stroma, as well as a lower lumen percentage, in hypertensive animals than in healthy rats (*p* < .05; Figure 4H–J). Exercise practice did not influence the proportion of epithelium in the prostatic acini (*p* > .05; Figure 4H), different from that observed for lumen and stroma proportion (*p* < .05; Figure 4I,J). The RT minimized the impact of PAH on the percentage of epithelium and lumen in the prostate tissue (p < .05; Figure 4H,I). The number of mast cells in the stroma of the ventral prostate was higher in hypertensive rats than in healthy animals (*p* < .05; Figure 5A–D,I). The practice of RT also increased the number of mast cells in the prostate of sedentary animals (*p* < .05; Figure 5I). The proportion of collagen was also higher in PAH animals than in healthy rats (*p* < .05; Figure 5E–H,J), with no effect of the RT protocol (*p* > .05; Figure 5J). However, the exercise did not contribute to minimizing the PAH effects on mast cell count and collagen percentage in the ventral prostate of hypertensive rats (*p* > .05; Figure 5I,J).

## 4 DISCUSSION

This study is a pioneer in revealing the detrimental effects of PAH on the ventral prostate morphophysiology. The monocrotaline‐induced PAH model in rats, although an exogenous insult, is widely recognized for mimicking the vasculopathy and lung remodelling observed in human PAH, regardless of its primary or secondary aetiology, and is particularly useful for investigating the molecular and cellular mechanisms underlying the disease. The results showed a reduction in ventral prostate biometry and antioxidant enzyme activity under hypertensive conditions, increasing the levels of oxidative metabolites in this relevant accessory sexual gland for male reproduction. PAH elicited alterations in the proportion of tissue components and the incidence of prostate pathologies. Our findings evidenced the modulating effects of the RT protocol in the ventral prostate of hypertensive rats. The practice of RT slightly acted in the PAH‐induced redox imbalance. However, exercise minimized the morphological and morphometric changes caused by the disease.

The hypertensive condition in rats led to low body weight and mass gain due to its catabolic effect.^34^ The reduced weight may be related to a high cardiac deficit and low metabolic efficiency caused by PAH.^35^ Other studies have associated PAH with body mass loss.^14^, ^36^, ^37^ Although RT improved body weight and mass gain in healthy animals, the same was not observed in PAH animals. The severity of the disease may have suppressed the anabolic effects of exercise. Likewise, PAH reduced the ventral prostate weight without affecting its relative weight. The first finding may be related to the role of PAH in inducing prostatic atrophy, potentially by reducing blood flow and hormonal levels as a consequence of PAH‐mediated vascular dysfunction. We recently showed that PAH decreases serum testosterone levels.^5^ Androgenic stimuli are crucial for prostate activity in producing protein fluids relevant to semen composition. Hence, impaired hormonal stimulus may lead to prostatic atrophy.^6^ Although healthy animals practising RT increased ventral prostate weight, exercise was not effective in protecting hypertensive rats against PAH‐induced prostate atrophy.

Herein, PAH significantly impaired the activity of antioxidant enzymes in the ventral prostate. The reduction in SOD and CAT activity is indicative of a state of exacerbated oxidative stress, common in cardiovascular diseases that may contribute to progressive tissue damage.^16^, ^38^ Low SOD levels may result in increased O_2_^·−^, which is highly reactive, while low CAT levels may not be efficient in transforming O_2_^·−^ into H_2_O_2_.^39^ In the broader scenario of PAH, endothelial dysfunction results either in or is a result of oxidative stress owing to ROS overproduction, accompanied by cell proliferation and inflammation.^16^, ^40^, ^41^ The altered activity of antioxidant enzymes in the prostate corroborates the idea of oxidative stress as one of the main pathological mechanisms resulting from PAH, even in non‐target organs. With the increase in ROS and free radicals, different signalling pathways are activated, culminating in an oxinflammation process. For instance, the presence of ROS can activate the nuclear factor erythroid‐2‐related factor (Nrf2) pathway, damaging the antioxidant defence systems by inhibiting the expression of antioxidant enzymes, such as SOD and CAT, and activating the nuclear factor kappa‐light chain‐enhancer of activated B cells (NF‐κB) pathway, related to the inflammatory response.^42^ Notably, PAH progression is associated with inflammation.^43^ The inflammatory process tries to repair tissue damage caused by oxidative stress, generating even more ROS and establishing a self‐perpetuating cycle.^44^ The same process occurred in the ventral prostate of PAH animals, once we observed stroma regions with intense inflammation, leukocyte marginalization and high mast cell count. The practice of RT was an adjuvant in the physiological process involved in the antioxidant balance in the ventral prostate of PAH rats. It increased the activity of the SOD enzyme, possibly improving the process of conversion of O_2_^·−^ into H_2_O_2_, less reactive than O_2_^·−^. The prostate is relatively sensitive to physical exercise.^45^ The relationship between physical exercise and the antioxidant system remains controversial.^46^, ^47^, ^48^ Given the nature of PAH, RT effects were not as effective, with a slight improvement in SOD enzymatic activity reflected in the tissue inflammatory process, reducing its stromal occurrence along with leukocyte marginalization.

Furthermore, NO levels were reduced in the ventral prostate of PAH‐monocrotaline‐induced rats. Besides being a marker of nitrosative stress, NO is a potent vasodilator in vascular diseases, acting as an antioxidant role in cellular signalling.^38^ Low NO levels contribute to the impairment of the general circulatory system under hypertensive conditions, delaying the transport of nutrients and ions relevant to the prostate's functioning. On the other hand, PAH rats exhibited high levels of MDA and CP in their prostate, confirming the hypothesis that oxidative stress plays a central role in the pathogenesis of PAH.^49^, ^50^ Lipid peroxidation caused by ROS attack compromises the structure of cell membranes, leading to apoptosis.^51^ Protein oxidation damages cells by disturbing protein functioning and cellular homeostasis.^52^ Herein, RT was not effective in maintaining adequate levels of NO in hypertensive rats, as well as reducing the pro‐oxidative environment. Despite the exercise being beneficial to improve SOD activity, it was not enough to reverse the oxidative state created by PAH.

The prostate histopathology revealed that PAH induced morphological changes, such as epithelial atrophy, stromal inflammation and leukocyte marginalization. These alterations are an inflammatory response to the pro‐oxidant process, eliciting tissue remodelling. Studies evaluating the ventral prostate in hypertensive animals have reported pathologies including intraepithelial neoplasia, cell proliferation in the stroma, expression of inflammatory proteins and an increase in prostatic pathologies.^8^, ^10^, ^11^, ^53^, ^54^ Interestingly, the RT practice was effective in reducing these pathologies. This finding highlights the potential of physical exercise to modulate inflammatory responses and prevent the progression of histological damage, even in the context of a chronic disease. Previous studies have described the relationship between exercise practice and the diminishing of the inflammatory process.^55^, ^56^

In the light of the foregoing, PAH affected morphometric and stereological parameters in the ventral prostate. The increase in epithelium and stroma proportion and low lumen percentage reflect a tissue remodelling process. Long‐term exposure to such hypertensive conditions may compromise glandular function and promote the development of more serious pathologies over time. The high proportion of stroma observed in PAH rats may be a consequence of fibrosis, characterized by the accumulation of collagen that hinders communication between the glandular compartment and the stroma, influencing the organ's inflammatory process.^57^ Moreover, the high epithelial proportion might indicate reactive epithelial hyperplasia in response to tissue damage driven by oxiinflammation.^58^, ^59^ However, this pathology was not observed in this study. Santos et al.^10^ reported that vascular diseases may cause epithelial prostatic hyperplasia. The increase in epithelium and stroma reduces the lumen proportion within the prostatic acini, impacting the gland secretory activity. The absence of this exacerbated epithelial growth may be related to the short period of PAH. The RT presented beneficial effects by increasing the lumen proportion and decreasing the stroma proportion in both healthy and hypertensive rats. This ability to preserve glandular histoarchitecture is important, suggesting that exercise may attenuate the progression of detrimental changes induced by PAH. However, RT did not influence the epithelium proportion and protect the ventral prostate against the induction of higher mast cell count and collagen deposition in the stroma, indicating that certain aspects of PAH‐induced tissue remodelling may not be altered by exercise.

## 5 CONCLUSION

This study showed that PAH can induce harmful changes in the ventral prostate, including glandular atrophy, oxidative stress and inflammation, culminating in tissue remodelling. This consequence can compromise prostate health and overall male reproductive function. RT practice showed beneficial effects, especially in reducing inflammation and preserving glandular architecture. However, this positive effect has limitations since exercise practice did not completely reverse PAH‐induced damage. Thus, RT may offer some protection for ventral prostate homeostasis under a hypertensive environment, but its efficacy is partial. Nevertheless, the action of physical exercise can be complemented by additional interventions, such as antioxidant and/or anti‐inflammatory strategies.

## AUTHOR CONTRIBUTIONS

**Luiz Otávio Guimarães‐Ervilha:** conceptualization, formal analysis, investigation, methodology, writing – original draft. **Thainá Iasbik‐Lima:** formal analysis, investigation. **Leôncio Lopes Soares:** conceptualization, investigation. **João Victor Leles Faria:** formal analysis. **Mariana Souza Oliveira:** formal analysis. **Renner Philipe Rodrigues Carvalho:** formal analysis. **Mônica Morais‐Santos:** formal analysis. **Antônio José Natali:** conceptualization, funding acquisition. **Mariana Machado‐Neves:** conceptualization, funding acquisition, project administration, supervision, writing – review and editing.

## FUNDING INFORMATION

This work was supported by Fundação de Amparo à Pesquisa do Estado de Minas Gerais (FAPEMIG; grant number PPM‐00621‐18; APQ‐00361‐23; BPD‐00843‐22; APQ‐02205‐24 to M.M.‐N.; and scholarship to L.O.G.‐E.) and Conselho Nacional de Desenvolvimento Científico e Tecnológico (CNPq; grant number 313524/2021‐1 to M.M.‐N; 151703/2024‐8 to L.O.G.‐E.).

## CONFLICT OF INTEREST STATEMENT

The authors declare that they have no known competing financial interests or personal relationships that could have appeared to influence the work reported in this paper.

## ETHICS STATEMENT

This study was carried out following the recommendations of the National Council for the Control of Animal Experimentation (CONCEA). All experimental procedures were reviewed and approved by the Animal Ethics Committee of the Federal University of Viçosa (CEUA‐UFV process number 38/2021). The authors are responsible for all aspects of this work, ensuring that good laboratory protocol practices and quality assurance methods were followed.

## Supporting information


Table S1.
