# Supplementary material for: Effect of pulmonary arterial hypertension on the morphology and antioxidant defence of the ventral prostate of sedentary and exercised rats
Source: Int J Exp Pathol. 2025 Oct 21;106(6):e70007. doi: 10.1111/iep.70007 (PMC12538510; doi:10.1111/iep.70007)
Supplement: Supplementary file 1 — Table S1. [file IEP-106-e70007-s001.docx]

**Supplementary Table 1.** ANOVA p-values

|  | Two-way ANOVA | | |
| --- | --- | --- | --- |
| Parameters | PAH effect | Exercise effect | Interaction |
| Body Weight (g) | 0.001 | 0.005 | 0.147 |
| Body Weight Gain (g) | 0.001 | 0.008 | 0.092 |
| Ventral prostate weight (g) | 0.006 | 0.004 | 0.966 |
| Relative weight of ventral prostate (mg/100g) | 0.643 | 0.002 | 0.777 |
| Superoxide dismutase (U/mg ptn) | 0.001 | 0.004 | 0.001 |
| Catalase (U/mg ptn) | 0.031 | 0.271 | 0.001 |
| Gluthatione-S-transferase (U/mg ptn) | 0.687 | 0.504 | 0.236 |
| Malondialdehyde (nmol/mg ptn) | 0.001 | 0.396 | 0.082 |
| Nitric oxide (µmol.mL^-1^) | 0.004 | 0.444 | 0.999 |
| Protein carbonylated (nmol.mL^-1^) | 0.012 | 0.294 | 0.524 |
| Epithelium (%) | 0.005 | 0.046 | 0.006 |
| Lumen (%) | 0.021 | 0.126 | 0.006 |
| Stroma (%) | 0.045 | 0.009 | 0.107 |
| Mast cell (mm²) | 0.029 | 0.015 | 0.901 |
| Collagen (%) | 0.006 | 0.975 | 0.031 |
